# Using mammographic density to predict breast cancer risk: dense area or percentage dense area

**DOI:** 10.1186/bcr2778

**Published:** 2010-11-18

**Authors:** Jennifer Stone, Jane Ding, Ruth ML Warren, Stephen W Duffy, John L Hopper

**Affiliations:** 1 Centre for Molecular, Environmental, Genetic and Analytical (MEGA) Epidemiology, The University of Melbourne, School of Population Health, Level 1, 723 Swanston St, Victoria, 3010, Australia; 2 Green-Templeton College, Oxford University, 43 Woodstock Road, Oxford, OX26HG, UK; 3 Department of Radiology, Box 218, Addenbrooke's Hospital, Cambridge, CB2 0QQ, UK; 4 Cancer Research UK Centre for Epidemiology, Mathematics and Statistics, Wolfson Institute of Preventive Medicine, Barts and The London School of Medicine and Dentistry, Queen Mary University of London, London, EC1 M 6BQ, UK

## Abstract

**Introduction:**

Mammographic density (MD) is one of the strongest risk factors for breast cancer. It is not clear whether this association is best expressed in terms of absolute dense area or percentage dense area (PDA).

**Methods:**

We measured MD, including nondense area (here a surrogate for weight), in the mediolateral oblique (MLO) mammogram using a computer-assisted thresholding technique for 634 cases and 1,880 age-matched controls from the Cambridge and Norwich Breast Screening programs. Conditional logistic regression was used to estimate the risk of breast cancer, and fits of the models were compared using likelihood ratio tests and the Bayesian information criteria (BIC). All *P *values were two-sided.

**Results:**

Square-root dense area was the best single predictor (for example, χ_1_^2 ^= 53.2 versus 44.4 for PDA). Addition of PDA and/or square-root nondense area did not improve the fit (both *P *> 0.3). Addition of nondense area improved the fit of the model with PDA (χ_1_^2 ^= 11.6; *P *< 0.001). According to the BIC, the PDA and nondense area model did not provide a better fit than the dense area alone model. The fitted values of the two models were highly correlated (*r *= 0.97). When a measure of body size is included with PDA, the predicted risk is almost identical to that from fitting dense area alone.

**Conclusions:**

As a single parameter, dense area provides more information than PDA on breast cancer risk.

## Introduction

A number of prospective, nested case control studies have shown that, for women of the same age, those with greater mammographic density are more likely to develop breast cancer [1]. Mammographic density refers to the white or opaque area on a mammogram representing the epithelial and stromal tissue in the breast. It can be measured many ways and usually is expressed as the percentage of dense area in the total area of the breast image. Denoted here as percentage dense area (PDA), it can be measured reliably within and between trained observers using a computerized thresholding technique [2], in part a consequence of PDA's having a large variance even after adjusting its mean for age.

Body mass index (BMI) is negatively correlated with PDA and accounts for almost one third of the variation in age-adjusted PDA [3]. When assessing PDA as a predictor of breast cancer risk, the model fit is improved if adjustment is made for BMI as well as for age [4]. That is, the association between PDA and breast cancer risk is negatively confounded by age and BMI; PDA decreases with age and with BMI, whereas breast cancer risk increases with each of these factors. (The positive association between breast cancer risk and BMI is in postmenopausal women. For premenopausal women, there is weak evidence of a negative association [5,6]).

When mammographic density is assessed using a computer-assisted thresholding technique, one obtains measures of the total area of the breast and the absolute area of density (dense area), and hence PDA (dense area divided by total area) and nondense area (total area minus dense area). BMI and weight are highly positively correlated with nondense area and PDA adjusted for age and BMI is highly positively correlated with dense area adjusted for age. This raises the question whether dense area or PDA is the better predictor of breast cancer risk.

The purpose of this study was to address this question using data from a large population-based, age-matched, case control study nested within cohorts of women attending mammographic screening programs in the United Kingdom.

## Materials and methods

A description of the study subjects has been reported previously [7] and is summarized below. Subjects were women attending mammographic screening at the UK National Health Service Breast Cancer Screening Program in Cambridge between November 1995 and August 2003 and in Norwich and Norfolk between March 1998 and March 2004. There was a combined total of 634 cases, and up to 3 controls (*N *= 1,880) were individually selected matched on screening centre, date of birth (within 6 mo), and date of screening (within 3 mo). The mean age was 57.4 years (range, 50-75 yr; SD, 4.7), and 67.5% of the cancers were screen-detected (as opposed to interval cancers).

Mammographic density was measured in the mediolateral oblique (MLO) view of the contralateral breast in cases and in the matching side in controls using a computer-assisted thresholding technique. This technique involves an observer's first selecting a gray value as a threshold to separate the image of the breast from the background. A second threshold is then selected to identify the edges of the mammographically dense tissues. The computer then records the number of pixels in the digitized image that lie within the defined areas. The result is a measure of the total area of the breast and a measure of dense area, which, when subtracted, gives the nondense area. PDA is thus defined as dense area divided by total breast area and expressed as a percentage. This method has been shown to be highly reliable, both within and between observers [8]. All of the mammograms were measured by the same observer (RMLW). A selection of 150 images was reanalyzed and used to determine the intraobserver agreement as calculated by an intraclass correlation coefficient (ICC). The measurement from the mediolateral oblique view was used in this analysis where the pectoral muscle was manually excluded prior to the measurement. Original mammograms were scanned using the Array 2905 DICOM ScanPro Plus Laser Film Digitizer version 1.3E software (Array Corp. Hampton, New Hampshire, USA) at absorbance of 4.7.

Other available mammographic measures were investigated for their association with breast cancer risk and were described in detail in a previous report [7] and thus are only briefly summarized here. Wolfe's classification is a visual assessment of mammographic density into four parenchymal patterns, N1, P1, P2 and DY, in increasing order of risk. Standard mammogram form (SMF) is a volumetric measurement of mammographic density generated using GenerateSMF version 2.2 β software from Siemens Molecular Imaging, Malvern, Pennsylvania, USA [9]. Finally, a visual assessment of mammographic density classified into 5% increments was estimated by a trained radiologist (RMLW).

This study was approved by the Norfolk Local Research Ethics Committee. This was a medical records study; therefore, direct consent from the patients was not required.

### Statistical analysis

Conditional logistic regression was used to estimate the associations between the mammographic measures (dense area, PDA and nondense area) and breast cancer risk in terms of odds ratios (ORs). ORs were estimated for quintiles (on the basis of the distribution of cases and controls combined) and as one-parameter log-linear functions of the measures treated as continuously distributed variables. For the latter, the most suitable scale was chosen by inspection of the OR estimates from a fitted one-parameter model plotted on the log scale against the medians of quintiles. All stated *P *values are two-sided. To test the relative goodness of fit, the likelihood ratio criterion was used for nested models and the Bayesian information criteria (BIC) for unnested models. BIC provide an asymptotic approximation to the negative log of the Bayesian marginal distribution under suitable regularity conditions, with the approximation being good for large *n *values [10]. In contrast to Akaike's Information Criterion (AIC), the BIC possess the advantageous property of asymptotic consistency, which ensures that as the sample size, *n*, grows to infinity, the BIC will almost surely select the true model, assuming it is amongst the set of those models under consideration. Practically, the larger penalty (as compared to AIC) often leads to better performance in settings where there are a small number of strong effects [11]. The smaller the BIC, the better the model fit. The exponential of the negative difference of any two BIC values can be interpreted as an approximate ratio of posterior probabilities of the two competing models. This provides an order of magnitude of how much more likely one model is the "true" model compared to the other, given the data.

## Results

**Figure 1:**
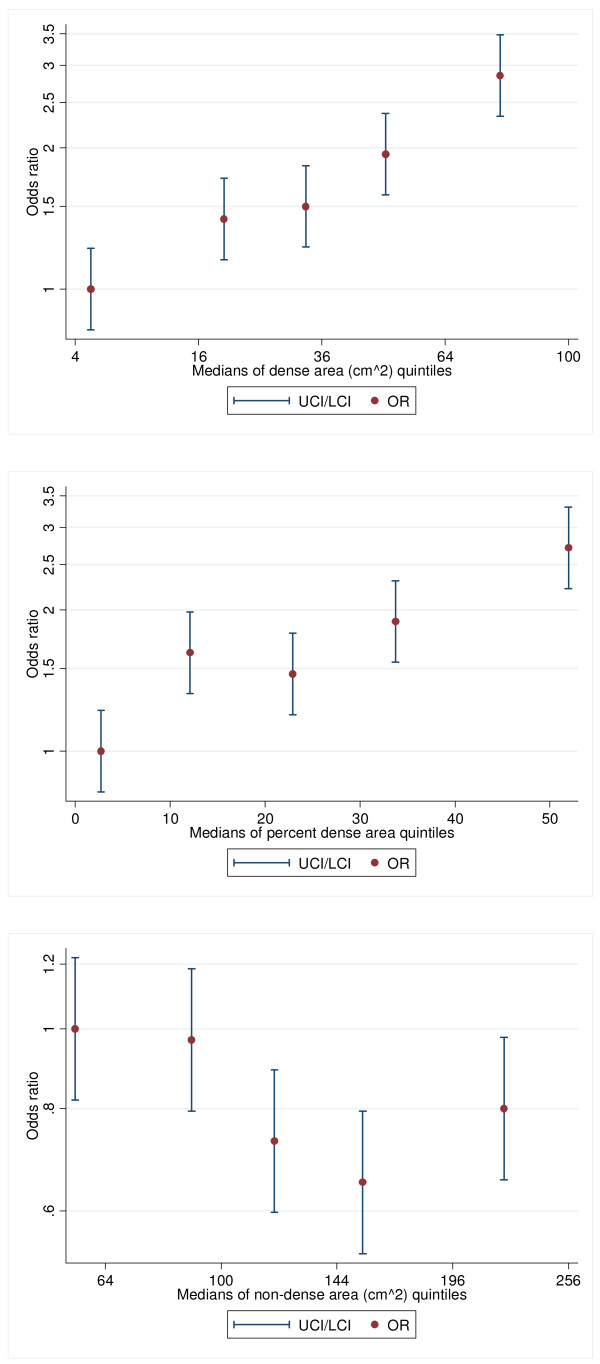
**Associations between each mammographic measure and breast cancer risk**. Odds ratios of the associations of quintiles of **(a) **dense area, **(b) **percentage dense area and **(c) **nondense area with breast cancer risk versus the medians of the quintiles of the corresponding mammographic measure.

The ICC estimates used to assess reader repeatability were 0.94 for dense area, 0.91 for percentage dense area and 0.96 for nondense area. Table 1 shows that the ORs increased with increasing dense area and PDA (both *P*_trend _< 0.001) and with decreasing nondense area (*P*_trend _= 0.02). The absolute mean difference between cases and controls was about one third of a standard deviation for both dense area and PDA and one eighth for nondense area. Table 1 also shows that when dense area was included in the model, the associations with PDA and nondense area were no longer significant (both *P*_trend _= 0.7). When the univariate OR estimates presented in Table 1 were plotted on the log scale against the quintile medians, a near-linear association was observed for dense area when it was square root-transformed and for PDA without being transformed. For nondense area, a square root transformation gave the closest to a linear fit (see Figure 1a-c). Therefore, in the analyses below fitting a one-parameter log-linear function, we used square root dense and nondense area and left PDA untransformed.

**Table 1:** Associations between quintiles (Q1-Q5) of the mammographic measures and breast cancer risk

						Adjusted for Dense Area		
						
Measurement	Cases (*n*)	Controls (*n*)	OR^b^	95% CI^c^	*P *value	OR	95% CI	*P *value
Dense area mean (SD^a^)	46.23 (34.07)	36.50 (29.36)						
Q1: 0.00-11.69 cm^2^	86	416	1.00	-	-	-		
Q2: 11.70-26.24 cm^2^	114	389	1.41	1.02-1.94	0.04	-		
Q3: 26.31-39.95 cm^2^	117	386	1.50	1.08-2.07	0.014	-		
Q4: 39.99-59.87 cm^2^	142	361	1.94	1.41-2.66	< 0.001	-		
Q5: 59.92-201.49 cm^2^	175	328	2.85	2.09-3.90	< 0.001	-		
*P *for trend					< 0.001			
Percentage dense area (PDA) Mean (SD)	29.30 (19.24)	23.81 (17.91)						
Q1: 0.00-7.01%	85	417	1.00	-	-	-	-	
Q2: 7.02-18.08%	126	377	1.62	1.18-2.21	0.003	1.32	0.78-2.21	0.3
Q3: 18.09-28.33%	115	388	1.46	1.06-2.01	0.022	0.96	0.52-1.75	0.9
Q4: 28.37-40.84%	136	367	1.89	1.38-2.60	< 0.001	1.02	0.54-1.91	0.95
Q5: 40.86-84.74%	172	331	2.71	1.98-3.71	< 0.001	1.25	0.65-2.40	0.5
*P *for trend					< 0.001			0.7
Nondense area mean (SD)	123.89 (65.79)	131.66 (64.28)						
Q1: 10.11-74.59	144	358	1.00	-	-	-	-	
Q2: 74.59-104.07	146	357	0.97	0.74-1.28	0.83	1.02	0.77-1.35	0.9
Q3: 104.14-134.70	116	387	0.73	0.54-0.98	0.04	0.83	0.62-1.13	0.2
Q4: 134.81-180.79	110	393	0.65	0.49-0.87	0.004	0.78	0.58-1.06	0.1
Q5: 181.07-456.33	118	385	0.80	0.60-1.08	0.15	1.08	0.79-1.49	0.6
*P *for trend					0.02			0.7

^a^Standard deviation; ^b^Odds ratio; ^c^Confidence interval.

Table 2 shows the OR associated with an increase of 1 standard deviation (SD) for each of the mammographic measures for various models as well as the -2 log likelihood (LL) and the BIC. Risk increased by 42% (95% confidence interval (CI), 29 to 56) per SD of square root dense area (*P *< 0.001), by 37% (95% CI, 25 to 50) per SD of PDA (*P *< 0.001) and decreased by 11% (95% CI, 2 to 19) per SD of square root nondense area (*P *= 0.01). The change in -2LL was greater for the dense area alone model than for the PDA alone model (53.22 versus 44.42).

**Table 2:** Associations between each continuously distributed mammographic measure and breast cancer

	OR^a ^Estimates (95% Confidence Interval)					
				
Measurement	DA^b^	PDA^c^	NDA^d^	*ΔΧ^2 ^*e	*P *value	BIC^f^
Base model (no predictors)	-	-	-	-		1663.78
Dense area (DA)	1.42 (1.29-1.56)	-	-	53.22	< 0.001	1618.39
Percent dense area (PDA)	-	1.37 (1.25-1.50)	-	44.42	< 0.001	1627.19
Nondense area (NDA)	-	-	0.89 (0.81-0.98)	5.96	0.01	1665.65
DA + NDA	1.42 (1.28-1.57)	-	1.00 (0.90-1.10)	0.01^g^	0.9^g^	1626.22
DA + PDA	1.31 (1.11-1.55)	1.10 (0.93-1.29)	-	1.18^g^	0.3^g^	1625.04
PDA + NDA	-	1.62 (1.41-1.85)	1.27 (1.11-1.46)	11.61^h^	< 0.001^h^	1623.41

^a^Odds ratio; ^b^OR per standard deviation (2.49); ^c^OR per standard deviation (17.91); ^d^per standard deviation (2.80); ^e ^Change in -2log likelihood (relative to base model and *P *values for model comparisons; ^f^Bayesian Information Criteria; ^g^Compared to dense area only model; ^h^Compared to percentage dense area only model.

Given dense area was in the model, addition of nondense area made no difference to the model fit (*P *= 0.9). When dense area and nondense area were fitted together, the dense area association was unchanged, whilst the association with nondense area collapsed completely (OR, 1.42; 95% CI, 1.28 to 1.57; and OR, 1.00; 95% CI, 0.90 to 1.10 for dense and nondense areas, respectively). That is, given dense area, knowledge of nondense area added nothing to the prediction of breast cancer risk.

When dense area and PDA were fitted together, the association with dense area was attenuated to 31% but remained significant (95% CI, 11 to 55), whilst the association with PDA attenuated substantially and was no longer significant (OR, 1.10; 95% CI, 0.93 to 1.29). That is, given dense area, knowledge of PDA did not improve the prediction of breast cancer risk.

When PDA and nondense area were fitted together, both factors were significant (OR, 1.62; 95% CI, 1.41 to 1.85; and OR, 1.27; 95% CI, 1.11 to 1.46; for percentage dense area and nondense area, respectively). The associations were greater in absolute strength than when fitted alone, but the estimates were positively correlated (*r *= 0.73). According to the BIC, the PDA and nondense area model did not provide a better fit than the dense area alone model (1623.41 versus 1618.39).

**Figure 2:**
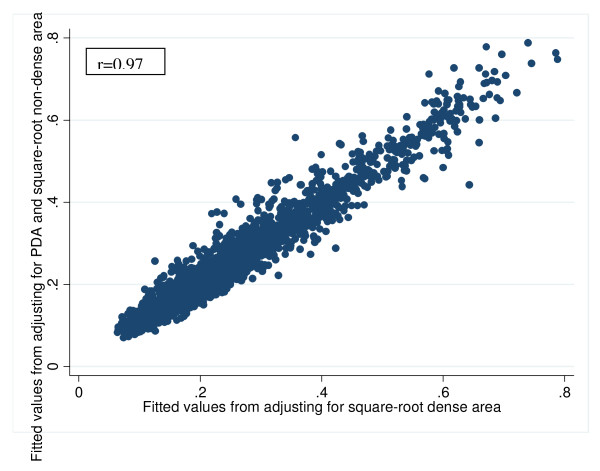
**Comparing breast cancer prediction models: percent dense area and nondense area versus dense area**. Fitted values from adjusting for percentage dense area and square root nondense area versus fitted values from adjusting for square root dense area.

Figure 2 shows that the fitted values from adjusting for PDA and nondense area were highly correlated with the fitted values from adjusting for dense area (*r *= 0.97). That is, although the absolute values of the associations with PDA and nondense area were increased when they were fitted together, for an individual woman the fitted model gives a risk prediction very similar to that from fitting a dense area alone model.

Other available mammographic measures (Wolfe classification, SMF and a visual assessment) were added separately to the dense area alone model and the PDA alone model, and none improved the model fit (data not shown). Both dense area and PDA remained significantly associated with breast cancer risk.

## Discussion

We found that both PDA and dense area were strongly associated with breast cancer risk; however, inclusion of dense area in a PDA-adjusted model improved the prediction of breast cancer risk, but not vice versa. This suggests that, in terms of a single parameter, dense area provides more information than PDA on breast cancer risk. PDA and nondense area also appeared to be strong predictors of breast cancer risk, but only in combination was the prediction as good as for dense area. Dense area and PDA adjusted for nondense area appear to be equivalent predictors of breast cancer risk. However, using only one predictor (dense area) provides a more parsimonious fit. Given that for the computer-assisted measurement used in this study the intraobserver reliability is the same for both dense area and PDA, dense area is arguably a better, simpler and more easily interpreted predictor of breast cancer risk.

Cases and controls were matched for age, so that all statements about risk estimates pertain to women of the same age. This was a large population-based study in which all of the mammograms were scanned and measured using the same machinery and by the same operator. The MLO view of the breast was measured as this view was the minimum recommended requirement for screening services in the UK and therefore the most readily available for all subjects. We have recently shown, using data from this same study sample, that there was no difference in the ORs or the fit of the models using the contralateral MLO or craniocaudal (CC) mammogram for dense area or percentage dense area [12]. This finding is consistent with that found by Vachon and colleagues [13], who showed no significant differences in the magnitude of associations of dense area or percentage dense area with breast cancer risk by side of cancer (ipsilateral versus contralateral) and mammogram view (MLO and CC), and there were essentially no differences in C-statistics between these models, indicating that the strength of the case control prediction for all of the models was the same. Therefore, we do not think that the results of this study or the generalizability of these findings are a function of the view that was measured.

Other breast cancer risk factor data were not available in this study, which is usually the situation for mammographic screening services. Outside of age and BMI, other known measured breast cancer risk factors account for very little of the variation in dense area or PDA [14] or breast cancer risk [3,15]. We used nondense area as a surrogate measure for weight or BMI. Nondense area represents the amount of fatty tissue in the breast, and it is highly correlated with BMI and weight and has been shown that 40% of the variation in nondense area can be explained by BMI [16]. Since there is a strong correlation (r = 0.97) between the estimates of association for PDA and for nondense area (that is, strong collinearity), the use of both terms in the same model calls for cautious interpretation; see 3rd paragraph below.

The risk of breast cancer in terms of a one-parameter model is well represented by both a log-linear function of square root dense area and a log-linear function of PDA. However, the former representation gave a better fit, and according to the BIC (which has the property that as the sample size increases, it will almost surely select the true model), the difference between the two one-parameter models is substantial enough to conclude that the dense area model is a better one-parameter representation. Furthermore, given the one-parameter dense area model, the addition of neither PDA nor nondense area produced a better fit, suggesting that these mammographic measures give little or no extra information on risk other than that which is contained in the dense area.

Adjusting for both PDA and nondense area simultaneously gave a better fit than PDA or nondense area alone, but according to the BIC, the one-parameter dense area model was still the better representation of the underlying true model. This information, combined with Figure 2 suggests that age-specific risk prediction associated with PDA after adjusting for nondense area is no better than fitting dense area alone.

As mentioned above, one has to be cautious when interpreting the simultaneous fit of PDA and nondense area. This study showed that nondense area (fatty tissue) acts very similar to that of BMI or weight in other reports [4]. We found that, like BMI and weight, nondense area was negatively correlated with PDA and negatively associated with breast cancer risk on its own. When fitted with PDA, in absolute terms, the regression coefficients for nondense area and PDA both became greater. But these estimates were correlated, so one cannot interpret them naively as if they were 'independent' and assume that their 'effects' were greater when fitted together. To derive the predicted risk for individual women from this two-parameter model fit, one needs to take into account that, on average, women with greater PDA will have lower nondense area. Hence, compared with when the covariates are fitted alone, what appears to be a greater risk gradient for a variable is offset by the apparently greater risk gradient for the other variable acting in the opposite direction. In any case, as shown by Figure 2 the predicted value for an individual woman from the two-parameter model is very close to those values from the one-parameter dense area alone model. In the context of the two-parameter model, interpreting the two risk estimates separately without understanding that they must be interpreted as a composite can lead to exaggerated claims about the strength of association of the individual components [4]. The estimates of association for PDA and dense area are also highly correlated when fitted together, and despite this the effect of dense area cancelled the other out.

Several other studies have found evidence of an association between dense area and breast cancer risk and have been summarized previously [14]. Overall, all of the previous studies have reported risk estimates similar in magnitude to that of percentage dense area. Maskarinec *et al*. [17] compared differences in percentage dense area and dense area to breast cancer risk incidence in populations at different risks of the disease and found that age-adjusted dense area may reflect breast cancer incidence better than percentage dense area. None of the studies provided risk estimates for nondense area.

In terms of applying the findings of this paper to a screening or research setting, there are issues about the measurement of dense area that need to be considered. Measurement of PDA has the advantage that it can be assessed reasonably well by eye by trained radiologists and has a natural scale (0% to 100%). This information could be used in a clinical setting much like Breast Imaging-Reporting and Data System assessment is used to alert the referring clinician that the ability to detect small cancers in the dense breast is reduced. Measurement of dense area involves measurement units that may differ according to a number of factors, including measurement technique and device. However, these issues are no different from the determination of, for example, bone mineral density (BMD) that has been standardized on the basis of age and sex, among other aspects, and used extensively for clinical and research purposes for many years. Similarly, dense area measures could be standardized for the population, and a woman's measure expressed in terms of number of standard deviations above or below the mean could be derived, as well as her predicted future breast cancer risk. Measurement of dense area is currently restricted to certain software packages as it cannot be assessed visually as PDA can. If it were automated, mammographic density could become a potentially useful adjunct to screening services and might lead to more cost-effective operation. The development of automated measures of mammographic density is currently ongoing, and the transition from film to digital mammography may aid this process.

There is also current activity dedicated to developing a volumetric measurement of mammographic density which would take into account the thickness of the dense tissue. In theory, measuring a three-dimensional risk factor from a three-dimensional rather than a two-dimensional image has the potential for obtaining more information on risk, but in this study the volumetric measurement method (SMF) was not found to be a better predictor than dense area or PDA of breast cancer risk [7].

## Conclusions

In summary, we found that dense area alone provides more information than PDA alone regarding age-specific breast cancer risk. When a measure of body size (or even a surrogate measure such as the area of nondense tissue in the breast) is included with PDA, the estimates are not independent and the predicted risk is almost identical to that from fitting dense area alone. Dense area is a simpler predictor of breast cancer risk which could help simplify breast cancer risk prediction models, which in turn could be used to optimize breast screening intervals. It could also help focus the search for environmental and genetic causes of mammographic density which will help us better understand the aetiology of breast cancer.

## Abbreviations

BMI: body mass index; ICC: intraclass correlation coefficient; MD: mammographic density; PDA: percentage dense area.

## Competing interests

The authors declare that they have no competing interests.

## Authors' contributions

RW, JD and SD were responsible for the conception, design and implementation of the study via their roles as a radiologist, research assistant and statistician, respectively. JD managed the collection and digitization of all of the mammograms. RW measured all of the mammograms. JS completed the statistical analysis and drafted the manuscript. JH and SD provided input on the statistical analysis and interpretation of the results. All authors commented on the draft manuscript and approved the final manuscript. All authors have read and approved the final submitted version of the paper.

## References

[B1] McCormack VA dos Santos Silva I Breast density and parenchymal patterns as markers of breast cancer risk: a meta-analysis Cancer Epidemiol Biomarkers Prev 2006 15 1159 1169 10.1158/1055-9965.EPI-06-0034 16775176

[B2] Yaffe M Boyd N Mammographic breast density and cancer risk: the radiological view Gynecol Endocrinol 2005 21 Suppl 1 6 11 10.1080/09513590400030053 16112949

[B3] Boyd NF Lockwood GA Byng JW Tritchler DL Yaffe MJ Mammographic densities and breast cancer risk Cancer Epidemiol Biomarkers Prev 1998 7 1133 1144 9865433

[B4] Boyd NF Martin LJ Sun L Guo H Chiarelli A Hislop G Yaffe M Minkin S Body size, mammographic density, and breast cancer risk Cancer Epidemiol Biomarkers Prev 2006 15 2086 2092 10.1158/1055-9965.EPI-06-0345 17119032

[B5] Hunter DJ Willett WC Diet, body size, and breast cancer Epidemiol Rev 1993 15 110 132 8405195 10.1093/oxfordjournals.epirev.a036096

[B6] Ursin G Longnecker MP Haile RW Greenland S A meta-analysis of body mass index and risk of premenopausal breast cancer Epidemiology 1995 6 137 141 10.1097/00001648-199503000-00009 7742399

[B7] Ding J Warren R Warsi I Day N Thompson D Brady M Tromans C Highnam R Easton D Evaluating the effectiveness of using standard mammogram form to predict breast cancer risk: case-control study Cancer Epidemiol Biomarkers Prev 2008 17 1074 1081 10.1158/1055-9965.EPI-07-2634 18483328

[B8] McCormack VA Highnam R Perry N dos Santos Silva I Comparison of a new and existing method of mammographic density measurement: intramethod reliability and associations with known risk factors Cancer Epidemiol Biomarkers Prev 2007 16 1148 1154 10.1158/1055-9965.EPI-07-0085 17548677 PMC2696797

[B9] Highnam R Pan X Warren R Jeffreys M Davey Smith G Brady M Breast composition measurements using retrospective standard mammogram form (SMF) Phys Med Biol 2006 51 2695 2713 10.1088/0031-9155/51/11/001 16723760

[B10] Schwarz G Estimating the dimension of a model Ann Stat 1978 6 461 464 10.1214/aos/1176344136

[B11] McQuarrie AD Tsai CL Regression and Time Series Model Selection 1998 Singapore: World Scientific

[B12] Stone J Ding J Warren RM Duffy SW Predicting breast cancer risk using mammographic density measurements from both mammogram sides and views Breast Cancer Res Treat 2010 124 551 554 10.1007/s10549-010-0976-y 20544272

[B13] Vachon CM Brandt KR Ghosh K Scott CG Maloney SD Carston MJ Pankratz VS Sellers TA Mammographic breast density as a general marker of breast cancer risk Cancer Epidemiol Biomarkers Prev 2007 16 43 49 10.1158/1055-9965.EPI-06-0738 17220330

[B14] Stone J Warren RM Pinney E Warwick J Cuzick J Determinants of percentage and area measures of mammographic density Am J Epidemiol 2009 170 1571 1578 10.1093/aje/kwp313 19910376

[B15] Vachon C Kuni CC Anderson K Anderson VE Sellers TA Association of mammographically defined percent breast density with epidemiologic risk factors for breast cancer (United States) Cancer Causes Control 2000 11 653 662 10.1023/A:1008926607428 10977110

[B16] Haars G van Noord PA van Gils CH Grobbee DE Peeters PH Measurements of breast density: no ratio for a ratio Cancer Epidemiol Biomarkers Prev 2005 14 2634 2640 10.1158/1055-9965.EPI-05-0824 16284389

[B17] Maskarinec G Pagano I Chen Z Nagata C Gram IT Ethnic and geographic differences in mammographic density and their association with breast cancer incidence Breast Cancer Res Treat 2007 104 47 56 10.1007/s10549-006-9387-5 17009106

